# Accuracy of malaria rapid diagnostic tests in community studies and their impact on treatment of malaria in an area with declining malaria burden in north-eastern Tanzania

**DOI:** 10.1186/1475-2875-10-176

**Published:** 2011-06-26

**Authors:** Deus S Ishengoma, Filbert Francis, Bruno P Mmbando, John PA Lusingu, Pamela Magistrado, Michael Alifrangis, Thor G Theander, Ib C Bygbjerg, Martha M Lemnge

**Affiliations:** 1 National Institute for Medical Research, Tanga Medical Research Centre, P.O Box 5004, Tanga, Tanzania; 2 Centre for Medical Parasitology at the Department of International Health, Immunology and Microbiology, University of Copenhagen and Department of Infectious Diseases, Copenhagen University Hospital (Rigshospitalet), Denmark

## Abstract

**Background:**

Despite some problems related to accuracy and applicability of malaria rapid diagnostic tests (RDTs), they are currently the best option in areas with limited laboratory services for improving case management through parasitological diagnosis and reducing over-treatment. This study was conducted in areas with declining malaria burden to assess; 1) the accuracy of RDTs when used at different community settings, 2) the impact of using RDTs on anti-malarial dispensing by community-owned resource persons (CORPs) and 3) adherence of CORPs to treatment guidelines by providing treatment based on RDT results.

**Methods:**

Data were obtained from: 1) a longitudinal study of passive case detection of fevers using CORPs in six villages in Korogwe; and 2) cross-sectional surveys (CSS) in six villages of Korogwe and Muheza districts, north-eastern, Tanzania. Performance of RDTs was compared with microscopy as a gold standard, and factors affecting their accuracy were explored using a multivariate logistic regression model.

**Results:**

Overall sensitivity and specificity of RDTs in the longitudinal study (of 23,793 febrile cases; 18,154 with microscopy and RDTs results) were 88.6% and 88.2%, respectively. In the CSS, the sensitivity was significantly lower (63.4%; χ^2 ^= 367.7, p < 0.001), while the specificity was significantly higher (94.3%; χ^2 ^= 143.1, p < 0.001) when compared to the longitudinal study. As determinants of sensitivity of RDTs in both studies, parasite density of < 200 asexual parasites/μl was significantly associated with high risk of false negative RDTs (OR≥16.60, p < 0.001), while the risk of false negative test was significantly lower among cases with fever (axillary temperature ≥37.5°C) (OR ≤ 0.63, p ≤ 0.027). The risk of false positive RDT (as a determinant of specificity) was significantly higher in cases with fever compared to afebrile cases (OR≥2.40, p < 0.001). Using RDTs reduced anti-malarials dispensing from 98.9% to 32.1% in cases aged ≥5 years.

**Conclusion:**

Although RDTs had low sensitivity and specificity, which varied widely depending on fever and parasite density, using RDTs reduced over-treatment with anti-malarials significantly. Thus, with declining malaria prevalence, RDTs will potentially identify majority of febrile cases with parasites and lead to improved management of malaria and non-malaria fevers.

## Background

Prompt and correct diagnosis is important for effective management of malaria cases in order to reduce morbidity and mortality caused by delayed or poor management of patients particularly among under-fives and pregnant women. Malaria diagnosis has for a long time, and particularly at community level, depended on clinical diagnosis (based on patients' history and symptoms), which has low specificity leading to over-diagnosis and over-treatment [1]. Thus, reliable diagnostic services for malaria are critical in order to reduce wastage of costly drugs, e.g. artemisinin combination therapy (ACT), and reduce drug selection pressure. The declining burden of malaria in some endemic countries [2,3], increases the risks of over-diagnosis and over-treatment even further.

Microscopic examination of Giemsa-stained blood smears confirms the presence of malaria parasites and remains the gold standard for malaria diagnosis despite its technical challenges and demand for trained personnel. However, most peripheral health facilities in malaria endemic countries lack the capacity to carry out parasitological diagnosis of malaria by microscopy [4-6]. In Tanzania, some hospitals which are commonly located in urban and few health facilities in rural areas have laboratories which are appropriately equipped and staffed to perform good quality malaria diagnosis by microscopy [7,8]. Following the recent recommendations of the World Health Organization (WHO) to adopt universal testing to confirm presence of malaria parasites (in patients of all age groups) before treatment with ACT, malaria rapid diagnostic tests (RDTs) are increasingly considered to be the best alternative in areas where high quality microscopy cannot be performed [9].

The RDTs utilize immuno-chromatographic methods to detect parasite specific antigens in lysed blood and the widely used tests detect either species-specific *Plasmodium falciparum *histidine rich protein 2 (PfHRP-2), Plasmodium lactate dehydrogenase (pLDH) or aldolase [10-12]. RDTs based on HRP-2 only detect *P. falciparum*, those based on pLDH and aldolase can detect *P. falciparum *and other species of human malaria [11], while some RDTs combine both HRP-2 and pLDH or HRP-2 and aldolase to enhance their accuracy for detection of all four species of malaria parasite that infect humans (*P. falciparum, Plasmodium vivax, Plasmodium ovale *and *Plasmodium malariae) *in mixed or mono-infections [13]. Currently, four brands of RDTs are registered by the Tanzania Food and Drug Authority (TFDA) and they include Paracheck Pf^® ^(Orchid Biomedical Systems - Mumbai, India), ParaHIT ^®^f (Span Diagnostics - Surat, India), ICT Malaria-Combo (ICT Diagnostics, South Africa) and SD Bioline Malaria Ag Pf/Pan (Standard Diagnostics Inc., India). Of these, Paracheck and ParaHIT are HRP-2 tests for detection of *P. falciparum*, while ICT Malaria-Combo and SD Bioline Malaria Ag Pf/Pan combine both HRP-2 and pLDH and can, therefore, detect *P. falciparum *and other human malaria parasite species.

Tanzania changed and implemented its malaria treatment guidelines to replace sulphadoxine/pyrimethamine (SP) with artemether/lumefantrine (ALu) as first-line drug for treatment of uncomplicated malaria in January 2007 [14]. The new guidelines recommend that ALu should be prescribed to all febrile children under five years of age suspected of malaria (irrespective of laboratory results) while treatment of individual aged ≥5 years of age has to be based on laboratory confirmation of malaria parasites (by microscopy or RDTs) except in health facilities without diagnostic facilities. This strategy was considered to be cost-effective when using ALu and in areas with moderate malaria transmission as shown by studies conducted in other malaria endemic areas [15,16]. Based on the current WHO recommendation of treating all patients after parasitological confirmation and lack of laboratory capacity to carry out malaria diagnosis in most of the health facilities in Tanzania, the Ministry of Health and Social Welfare through the National Malaria Control Programme (NMCP) plans to introduce a new malaria diagnostic policy in the country aiming at using RDTs in places without facilities for microscopy.

Currently, the NMCP in Tanzania has embarked on operational research in order to identify key issues which need to be addressed before, during and after deployment of RDTs including choice and cost of RDTs, setting up systems for quality assurance and others such as issues related to procurement of the tests [1]. Although RDTs require minimal skills and are easy to read, which allow them to be used by moderately trained health workers, their accuracy (sensitivity and specificity), storage under field condition and application for treatment of malaria remain a challenge [11,12,17-21]. This study was conducted in an area with declining burden of malaria to assess; 1) the accuracy of RDTs when used at different community settings; 2) the impact of RDTs on anti-malarial dispensing when used by village helpers known as community-owned resource persons (CORPs); and 3) adherence of CORPs to treatment guidelines by treating attended cases based on RDT results. Two different community study set-ups were used; a longitudinal study using CORPs to passively monitor febrile illness, and cross-sectional surveys (CSS) to monitor the declining burden of malaria in areas, which were until recently holo/hyper-endemic to malaria in north-eastern Tanzania.

## Methods

### Study site and design

Data on malaria diagnosis performed using RDTs and related information were obtained from two ongoing studies in six villages in Korogwe and four in Muheza districts, in Tanga region, north-eastern Tanzania. The first study was a longitudinal follow-up to passively detect fever episodes using CORPs in the villages of Kwamasimba, Mkokola, Kwamhanya, Magundi, Kwashemshi and Mg'anza in Korogwe district and covered a period of 4.5 years from January 2006 to June 2010. The six villages in Korogwe are under the demographic and health surveillance system (DHSS) which together with CORPs activities allow longitudinal follow-up of patients treated at by CORPs in these communities. Two of the six villages in Korogwe district (Kwamasimba and Mkokola) have been involved in different studies on immuno-epidemiology of malaria since 2003 and four more villages (Kwamhanya, Magundi, Kwashemshi and Mg'aza) were enrolled in similar studies in 2005. Detailed description of these villages has been published previously [22,23]. The second study involved CSS conducted in six villages between May 2007 and June 2010 including the two villages in Korogwe (Kwamasimba and Mkokola which also participated in the longitudinal study) and four in Muheza district which participated in different studies (Magoda and Mamboleo from 1992, Mpapayu from 1997 [24,25] and Mgome which was enrolled in other studies in 2001 [26,27]). In these areas, malaria burden has declined in recent years as reported by Mmbando *et al *[28] and Ishengoma *et al *(unpublished data).

### Data collection methods

#### Longitudinal study

Cases presenting to CORPs with history of fever within 48 hours before the visit had history taken and clinical presentation assessed. Axillary temperature was measured from all patients using a digital thermometer to obtain fever at presentation, which was defined as axillary temperature ≥37.5°C. Recruitment and initial training of CORPs was conducted as previously reported [22,28]. One of the key elements of the training given to CORPs was instructions on how to identify patients to treat and those who needed referral to the nearby health facility. Before introduction of ALu and RDTs in February 2007, CORPs were further trained on how to use modified standard operating procedures (SOPs) written in Kiswahili (the Tanzanian national language) with simple guidelines on how to perform RDTs for malaria parasite detection, interpret the results and administer treatment to malaria patients with ALu.

The longitudinal study has been divided into pre-RDT (from January 2006 to January 2007) and post-RDT periods (February 2007 to June 2010). During the pre-RDT period, all patients with history of fever or fever at presentation were treated with SP. Thick and thin blood smears were prepared from finger prick blood samples for malaria diagnosis by microscopy that was performed later in the laboratory. In the post-RDT period, thick and thin blood smears were also collected as above and RDTs for malaria diagnosis was performed by CORPs. According to the SOPs, all children below five years of age (with history of fever or fever at presentation) were supposed to be treated with ALu regardless of RDT results while those aged ≥5 years were only treated with ALu if they had positive RDTs. Children and infants weighing less than 5Kg were referred to the nearest health facility for treatment. Routine supervision was conducted weekly during both periods by a medical doctor or a trained assistant medical officer and a laboratory technician. During the supervisory visits, general performance of CORPs was assessed and on-spot training support provided. Blood smears prepared by CORPs were collected during weekly supervision, and brought to the laboratory for microscopic examination by trained laboratory technicians. The results of microscopic examination of blood smears were given back to CORPs during the next supervision and were used for treatment of cases not given anti-malarials due to negative RDT results.

#### Cross-sectional surreys (CSS)

Five malariometric surveys were conducted either during short (November/December) or long (April/June) rainy seasons from May 2007 to June 2010. About 250 study participants were randomly selected from each of the study villages with the aim of recruiting 120 children up to five years of age (20 in each age band of one year, from 0 - 5.9 years), 100 children from six to 15.9 years (10 from each age band of two years) and 30 from 16 to 19.9 years (15 individuals from each of the age bands of 16 - 17.9 and 18-19.9 years). Each recruited person was examined by a medical doctor and axillary temperature was measured using a digital thermometer. Measured fever (fever at presentation) was defined as axillary temperature ≥37.5°C. Blood samples were collected from each study participant by venous bleeding or finger prick by trained laboratory technicians/research nurses for parasitological examination and other laboratory analyses. Blood smears for detection of malaria parasites were prepared by trained laboratory technicians, dried in the field and later brought to the laboratory for further processing. Detection of malaria parasites by RDTs was performed on fresh samples and participants with fever or other malaria related symptoms, and positive RDTs results were treated using ALu and paracetamol. Testing by RDTs was done by trained laboratory technicians/research scientists according to manufacturers' instructions.

### Laboratory analysis

In the laboratory, blood smears were stained using 10% Giemsa solution for 30 minutes and examined under high power objective. In positive smears, asexual and sexual parasites were counted against 200 and 500 white blood cells (WBCs), respectively. Parasite density was obtained by multiplying the parasite counts by 40 for asexual and 16 for sexual parasites (assuming each micro litre of blood contained 8000 WBCs). A smear was declared negative after examining 200 high power fields. For quality control purposes, each blood smear was examined by two technicians blinded of the patient status and RDT results. The final parasite density was taken as the average of the counts of the two technicians if their results did not differ by more than 50% for blood smears with ≥400 asexual parasites/μl of blood. In blood smears with < 400 asexual parasites/μl, any counts of each of the two technicians was accepted and used to calculate the average parasite density. Blood smears with discordant results were re-examined by a third technician and the results of any two technicians was accepted as explained above. Further discordant smears were resolved by a team of three technicians who re-examined such smears at the same time.

Two different types of HRP-2 based RDTs were used in the two studies; ParacheckPf^® ^(Orchid Biomedical Systems - Mumbai, India) was used in the longitudinal study from November 2007 to June 2009 while ParaHIT^®^f (Span Diagnostics - Surat, India) was used from February to October 2007 and July 2009 to June 2010. In the CSS, Paracheck was used in November 2007 and May 2008 while ParaHIT was used in May 2007, 2009 and 2010. For quality control (QC) purposes, all RDTs were stored at 4 - 8°C in a special air-conditioned room, which was monitored twice a day during working days and once on week-ends and public holidays. To avoid keeping large number of RDT kits in the villages without appropriate storage rooms, small batches were delivered to the CORPs during weekly supervision. The RDTs delivered to CORPs were stored at ambient temperature (temperature ranged from 25 to 34°C) and the duration of storage in the field was kept at less than two weeks.

### Ethical considerations

The studies which contributed data used in this paper were approved by the Medical Research Coordination Committee of the National Institute for Medical Research. Verbal and written informed consent was sought from patients or parents/guardians in case of children. For both studies, village meetings were held to explain and discuss the study plans with community members. In case of CSS, feedback and results of the previous surveys were communicated back to the communities through the above meetings together with a written report.

### Data analysis

Data management was done using Microsoft Access database with double entry, validation and cleaning; followed by analysis using STATA version 10 (STATA Corp Inc., TX, USA) and R - Statistical Software [29]. Sensitivity and specificity of RDTs were calculated by comparing RDT results with microscopy as a gold standard. Predictors of a risk of obtaining false negative RDTs results as determinants of sensitivity of RDTs were calculated using a multivariate logistic regression model adjusting for age of study participants (under-fives *vs*. cases aged ≥5 years old), fever status at presentation (fever was defined as axillary temperature ≥37.5°C), parasite density and year of study as a measure of malaria parasite prevalence. For predictors of a risk of obtaining false positive RDT results as determinants of specificity of RDTs, adjustment was done for age of study participants, fever status and year of study. Since the villages included in this study had different malaria transmission intensity, which depends on altitude [23], adjustment was made in the regression model to account for the differences in transmission intensity by grouping the villages into those from either low or high transmission areas. The villages of Kwamasimba, Kwamhanya and Magundi (located in the highlands) were categorized into low transmission while Kwashemshi, Mkokola and Mng'aza in Korogwe, and Magoda, Mamboleo, Mgome and Mpapayu in Muheza district (located in the lowlands) were considered to be in high transmission areas. P-value < 0.05 was considered to be significant.

## Results

### Baseline characteristics

In the longitudinal study, a total of 23,793 febrile cases were attended by CORPs between January 2006 and June 2010. A majority of these (18,217, 76.6%) were attended during the post-RDT period (February 2007 - June 2010) while 5,576 (23.3%) cases were attended in the pre-RDT period (January 2006 - January 2007). Baseline characteristics of the cases attended by CORPs are shown in table 1. Most of the cases attended in the entire period of the study (> 77.6%) were aged over 5 years. The mean age of attended cases was significantly higher during the pre-RDT period compared to the post-RDT period (t = 12.3, p < 0.001). Almost all patients (99.0% and 99.4% during the pre- and post-RDTs periods, respectively) had a history of fever (within 48 hours before reporting to the CORPs) but only 31.9% (30.9%, and 32.2% during the pre and post-RDTs periods, respectively) had fever (≥37.5°C) at presentation to CORPs. Malaria parasite prevalence was similar in the two periods while geometric mean parasite density were significantly higher during the post-RDT compared to the pre-RDT period (t-test = 4.9, p < 0.001) (Table 1). Out of 18,158 patients tested for malaria parasites using RDTs, only 27.8% were found to be positive (23.0% in children below 5 years of age and 29.2% in those aged ≥5 years).

**Table 1:** Baseline characteristics of participants enrolled in the longitudinal study and CSS in Korogwe and Muheza districts

Variable	Longitudinal study (Pre-RDTs)	Longitudinal study (Post-RDTs)	CSS
Number of cases sampled/attended	5576	18217	5759
Mean age in years (range)	26.2(0, 96.0)	22.2(0, 98.2)	8.1(0, 20.0)
Age group (< 5 years, %)	1065(19.1)	4275(23.5)	2033(35.3)
Sex- Male (%)	42.9	46.0	45.4
Cases with fever (axillary temperature ≥37.5°C, %)	1720(30.9)	5866(32.2)	176(3.1)
Cases with BS positive (%)	1130(20.5)	3779(20.8)	1045(18.2)
Cases wtih RDT Positive (%)	NA	5038(27.8)	931(16.2)
PfGMPD* (95% CI)	3589(3081,4180)	5479(5051,5944)	489(428,558)

CSS = cross-sectional surveys, % = percentage,°C = degree centigrade, BS = blood smears, RDTs = malaria rapid diagnostic tests, *PfGMPD = *Plasmodium falciparum *geometric mean parasite density (asexual parasites/μl), 95% CI = 95% confidence interval, NA = Not applicable

Five CSS were conducted in a period of 4 years (from May 2007 to May 2010) and all were done during the long rainy season in May except one survey, which was conducted during the sort rainy season in November/December 2007. In total, 5,759 individuals aged 0-20 years (mean = 8.1, SD = 5.1 years) participated in the surveys and most of them (64.7%) were aged between 5 and 20 years (Table 1). Individuals with history of fever (within 48 hours) accounted for 13.5% while only 3.1% had fever at presentation (≥37.5°C). Overall malaria parasite prevalence by microscopy (in all age groups) was 18.2% (ranged from 29.6% in 2008 to 10.4% in 2010) and 16.2% were positive by RDTs (range: 28.9% in 2008 to 6.4% in 2010). Compared to the longitudinal study, participants enrolled in the CSS had lower geometric mean parasite density (Table 1). In both studies, cases with fever (≥37.5°C) were more likely to have malaria parasites compared to those without fever, after adjusting for age, year of study and level of transmission intensity (in the longitudinal study, adjusted OR = 3.00, p < 0.001 and in the CSS, adjusted OR = 4.16, p < 0.001).

### Sensitivity and specificity of RDTs

In the longitudinal study, 8,848(48.6%) cases were tested with Paracheck and the rest (51.4%) were tested with ParaHIT while in the CSS, 2,664 (46.3%) individuals were tested with Paracheck and 3,195 (53.7%) were tested with ParaHIT. The number of cases with false positive RDT results (with negative blood smear results by microscopy) in the longitudinal study was higher (1,695, 9.3%) compared to those with false negative RDTs (427, 2.4%). Paracheck had large number of cases with false positive RDTs (1,008, 11.4%) compared to ParaHIT (687, 7.4%) while the proportion of cases with false negative results was similar for the two brands of RDTs (2.2% for Paracheck and 2.5% for ParaHIT). In the CSS, 268 (4.7%) individuals had false positive results while 382 (6.6%) had false negative results. Among individuals tested with Paracheck in the CSS, 161(6.1%) had false positive results by RDTs and 164(6.2%) had false negative results while for ParaHIT, 107(3.5%) had false positive and 218(7.0%) had false negative results corresponding to the high specificity but low sensitivity of ParaHIT observed in the cross-section surveys.

**Figure 1:**
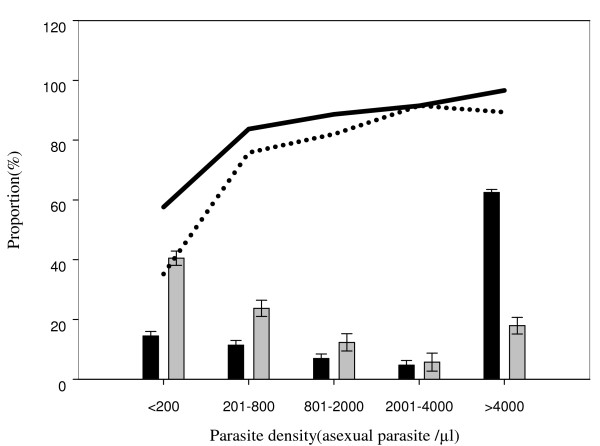
**Overall sensitivity of RDTs by parasite density for blood smear positive samples in the longitudinal study and CSS in Korogwe and Muheza districts**. Bars represent proportion of cases with positive blood smear results by different categories of parasite density, asexual parasites/μl (black bars = longitudinal study, n = 3793; and grey bars = CSS, n = 1045); Solid line = sensitivity of RDTs in the longitudinal study and dotted line = sensitivity of RDTs in the cross-sectional surveys (CSS)

**Figure 2:**
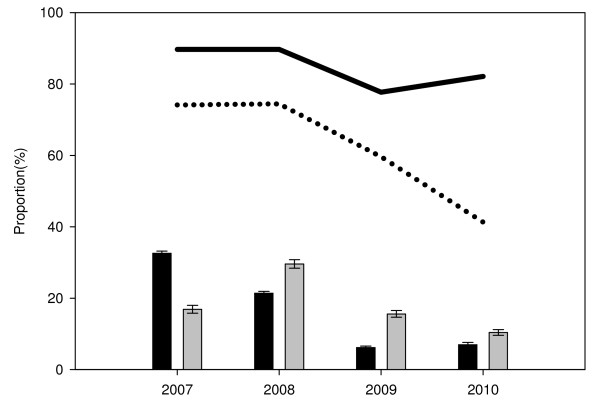
**Sensitivity of RDTs by malaria parasite prevalence stratified by the year of study**. Bars represent proportion of cases with positive blood smear results by microscopy by year of study from 2007 to 2010 (black bars = longitudinal study and grey bars = cross-sectional surveys - CSS); Solid line = sensitivity of RDTs in the longitudinal study and dotted line = sensitivity of RDTs in the cross-sectional surveys (CSS)

Using the results of microscopic examination of Giemsa-stained blood smears as gold standard, the overall sensitivity of RDTs was significantly higher in the longitudinal study (88.6%) compared to the CSS (63.4%, χ^2 ^= 367.7, p < 0.001). For both studies, the sensitivity was higher in febrile patients irrespective of age group (Table 2). Both RDT brands had similar sensitivity in the longitudinal study (adjusted OR = 1.03, p = 0.896) while in the CSS, ParaHIT (46.3%) had significantly lower sensitivity compared to Paracheck (74.3%, adjusted OR = 8.63, p < 0.001) after correcting for age of individuals tested, fever, parasite density, parasite prevalence and transmission intensity (Table 3). In both studies, the sensitivity of RDTs increased with parasite density, ranging from a low 35.2% at < 200 asexual parasites/μl to 96.6% at parasite density ≥4001 asexual parasites/μl (Table 3 and Figure 1). Parasite density < 200 asexual parasites/μl was significantly associated with high risk of false negative RDT results (adjusted OR≥16.60, p < 0.001) while the risk of false negative test was significantly lower among cases with fever (adjusted OR ≤ 0.63, p ≤ 0.027) (Table 3). For both studies, the sensitivity of RDTs decreased with decreasing malaria parasite prevalence, which occurred over the 3.5 years of the study (Table 3 and Figure 2). The effect of malaria transmission intensity on the risk of false negative RDT results varied across the years and geographical location of the study villages with higher sensitivity during years with high parasite prevalence in 2007 and 2008, and in villages with relatively high malaria transmission in the CSS only (Table 3).

**Table 2:** Sensitivity and specificity of RDTs in the longitudinal study and CSS in Korogwe and Muheza districts

		Positive rate			
Study type	Category (n)	Microscopy (%, 95% CI)	RDTs (%, 95% CI)	Sensitivity (%, 95% CI)	Specificity (%, 95% CI)
Longitudinal study	Under-fives + fever (n = 2116)	27.8 (25.9-29.7)	31.9(29.9-33.9)	92.1(89.9-94.3)	91.3(89.9-92.7)
	Under-fives, no fever(n = 2159)	12.1(10.7-13.5)	14.3(12.8-15.8)	85.4(81.1-89.7)	95.6(94.7-96.5)
	Above 5 years + fever (n = 3750)	37.3(36.0-39.0)	48.6(47.0-50.2)	92.3(90.9-93.1)	77.5(75.8-79.8)
	Above 5 years, no fever (n = 10186)	15.0(14.3-15.7)	22.1(21.3-22.9)	84.6(82.8-86.4)	89.0(88.2-89.6)
	Overall (18213)	20.8(20.2-21.4)	27.8(27.1-28.5)	88.6(87.5-89.7)	88.2(87.7-88.7)
CSS	Under-fives + fever (n = 83)	32.5(22.4-42.6)	41.0(30.4-51.6)	96.3(89.0-100.0)	85.7(76.5-94.9)
	Under-fives, no fever(n = 1940)	8.0(6.8-9.2)	10.5(9.1-11.9)	66.7(59.3-74.1)	94.4(93.3-95.5)
	Above 5 years + fever (n = 96)	40.9(31.1-50.7)	41.9(32.0-51.8)	89.5(79.8-99.2)	90.9(83.3-98.5)
	Above 5 years, no fever (n = 3906)	22.6(21.3-23.9)	18.0(16.8-19.2)	60.5(57.1-63.9)	94.5(93.7-95.3)
	Overall (n = 5759)	18.2(17.2-19.2)	16.2(15.2-17.2)	63.4(59.8-67.1)	94.3(93.6-95.0)

CSS = Cross-sectional surveys, RDT = malaria rapid diagnostic tests, % = percentage,°C = degree centigrade, 95% CI = 95% confidence interval

**Table 3:** Predictors of a risk of obtaining a false negative RDT results among individuals with positive blood smears as determinants of RDT sensitivity in the longitudinal study and CSS in Korogwe and Muheza districts

	Longitudinal study			CSS		
Variable/covariate	Sensitivity (%)	Unadjusted OR(p-value)	Adjusted OR(p-value)	Sensitivity (%)	Unadjusted OR(p-value)	Adjusted OR(p-value)
**RDT type**						
Paracheck	1422/1616(88.0)	reference	reference	475/639(74.3)	reference	reference
Parahit	1919/2152(89.2)	0.89(0.259)	1.03(0.896)	188/406(46.3)	3.36(< 0.001)	8.63(< 0.001)
**Age group**						
< 5 years	761/845(90.1)	reference	reference	130/184(70.7)	reference	reference
≥5 years	2580/2923(88.3)	1.20(0.148)	0.65(0.005)	533/861(61.9)	1.48(0.026)	0.98(0.682)
**Fever **(axillary temperature ≥37.5°C)						
No fever	1514/1787(84.7)	reference	reference	596/969(61.5)	reference	reference
With fever	1826/1980(92.2)	0.47(< 0.001)	0.63(< 0.001)	60/65(92.3)	0.13(< 0.001)	0.32(0.027)
**Pf Density**(asexual parasites/μl)						
≥4001	2274/2355(96.6)	reference	reference	167/187(89.3)	reference	reference
2001-4000	162/177(91.5)	2.60(0.001)	2.465(0.001)	54/59(91.5)	0.77(0.623)	0.84(0.752)
801-2000	232/262(88.6)	3.63(< 0.001)	3.73(< 0.001)	105/128(82.0)	1.83(0.067)	1.77(0.104)
201-800	359/429(83.7)	5.47(< 0.001)	5.21(< 0.001)	188/248(75.8)	2.66(< 0.001)	2.00(< 0.001)
< 200	314/545(57.6)	20.65(< 0.001)	21.17(< 0.001)	149/423(35.2)	15.36(< 0.001)	16.60(< 0.001)
Year*						
2007	1912/2132(89.7)	reference	reference	147/201(74.1)	reference	reference
2008	1141/1272(89.7)	0.99(0.985)	1.19(0.510)	326/438(74.4)	0.98(0.936)	1.85(0.013)
2009	192/247(77.7)	2.45(< 0.001)	2.63(< 0.001)	122/246(49.6)	2.91(< 0.001)	0,72(0.198)
2010	96/117(82.1)	1.90(0.011)	3.91(< 0.001)	66/160(41.3)	4.08(< 0.001)	0.99(0.998)
Location**						
Highland	1084/1199(90.4)	reference	reference	22/51(43.1)	reference	reference
Lowland	2257/2569(87.9)	1.20(0.026)	1.10(0.441)	641/994(64.5)	0.41(0.003)	0.19(< 0.001)

RDT = malaria rapid diagnostic tests, CSS = cross-sectional surveys, OR = odds ratio, Pf = *Plasmodium falciparum*

*Year of study was used to represent malaria parasite prevalence in both studies.

**Geographical location of the village based on altitude indicating level of malaria transmission

**Figure 3:**
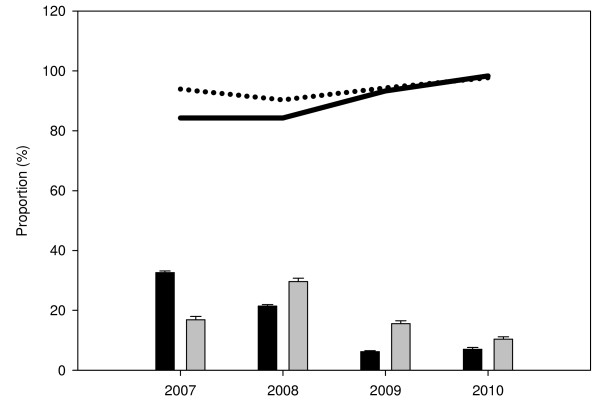
**Specificity of RDTs by malaria parasite prevalence stratified by the year of study**. Bars represent proportion of cases with positive blood smear results by microscopy by year of study from 2007 to 2010 (black bars = longitudinal study and grey bars = cross-sectional surveys - CSS); Solid line = specificity of RDTs in the longitudinal study and dotted line = specificity of RDTs in the cross-sectional surveys (CSS)

The overall specificity of RDTs was significantly higher in the CSS (94.3%) compared to the longitudinal study (88.2%, χ^2 ^= 143.1, p < 0.001) and in cases without fever compared to those with fever (Table 2). The risk of false positive RDT results was significantly lower with ParaHIT compared to Paracheck (adjusted OR ≤ 0.53, p < 0.001). For both studies, the specificity of RDTs increased with decreasing malaria parasite prevalence (Table 4 and Figure 3). The risk of false positive RDT results as determinants of specificity was significantly higher in cases with fever compared to afebrile cases (adjusted OR≥2.40, p < 0.001). Decreasing malaria parasite prevalence had significantly lower risk of false positive RDT results in the longitudinal study (OR ≤ 0.57, p < 0.001) compared to the CSS where the risk was significantly lower in 2010 only (OR = 0.38, p < 0.001) (Table 4).

**Table 4:** Predictors of a risk of obtaining false positive RDT results among individuals with negative blood smears as determinants of RDT specificity in the longitudinal study and CSS in Korogwe and Muheza districts

Variable/covariate	Longitudinal study			CSS		
	Specificity (%)	Unadjusted OR(-value)	Adjusted OR(p-value)	Specificity (%)	Unadjusted OR(-value)	Adjusted OR(p-value)
**RDT type**						
Paracheck	6216/7226(86.0)	reference	reference	1857/2018(92.0)	reference	reference
Parahit	6475/7164(90.4)	0.65(< 0.001)	0.48(< 0.001)	2580/2687(96.0)	0.40(< 0.001)	0.53(< 0.001)
**Age group**						
< 5 years	3119/3441(93.6)	reference	reference	1738/1847(94.1)	reference	reference
≥5 years	9497/10979(86.5)	2.30(< 0.001)	2.86(< 0.001)	2699/2858(94.4)	0.93(0.625)	1.01(0.935)
**Fever **(axillary temperature ≥37.5°C)						
No fever	9492/10555(90.1)	reference	reference	4309/4563(94.4)	reference	reference
With fever	3198/3856(82.9)	1.88(< 0.001)	2.40(< 0.001)	98/111(88.3)	2.25(0.007)	2.84(0.001)
Year*						
2007	3707/4396(84.3)	reference	reference	917/977(93.9)	reference	reference
2008	3944/4678(84.3)	1.00(0.982)	0.57(< 0.001)	940/1041(90.3)	1.64(0.003)	0.97(0.850)
2009	3502/3753(93.3)	0.38(< 0.001)	0.28(< 0.001)	1230/1305(94.3)	0.93(0.693)	0.99(0.955)
2010	1537/1563(98.3)	0.09(< 0.001)	0.10(< 0.001)	1350/1382(97.7)	0.36(< 0.001)	0.38(< 0.001)
Location**						
Highland	4642/5381(86.3)	reference	reference	1321/1340(98.6)	reference	reference
Lowland	8049/9009(89.3)	0.75(< 0.001)	0.74(< 0.001)	3116/3365(92.6)	5.55(< 0.001)	7.33(< 0.001)

RDT = Malaria rapid diagnostic tests, CSS = cross-sectional surveys, OR = odds ratio

*Year of study was used to represent malaria parasite prevalence in both studies.

**Geographical location of the villages based on altitude indicating level of malaria transmission

### Impact of prompt diagnosis using RDTs on malaria treatment

All cases attended during the pre-RDTs period (5,576) except two had blood smears taken and 5,513(98.9%) had microscopic results, whereby 20.5% had malaria parasites detected later in the laboratory by microscopy. Information regarding treatment was available for 5,557 (99.7%) and 5,478 cases (98.6%; including 97.2% in under-fives and 98.9% in cases aged ≥5 years) were treated with SP, which was the first-line drug for treatment of uncomplicated malaria (Table 5). During the post-RDTs period, which involved 18,217 cases, all except four cases had blood smears results and 20.8% had malaria parasites detected by microscopy (Table 5). Results for both microscopy and RDTs were available for 18,154 (99.7%) cases and 18,090(99.3%) had treatment records. Treatment with ALu was given to 8,562 (47.3%), whereby 96.6% of the under-fives and 32.1% of those aged ≥5 years were treated irrespective of RDT results. Based on RDT results, only 4,893 (27.1% of all cases tested with RDTs) were treated with positive RDTs while 3,629 (20.1%) were treated with ALu despite negative RDT results. Among patients treated with negative RDTs in the different age groups, 3,154 (74.3%) were under-fives and 475 (3.4%) were aged ≥5 years (Table 5). In the post-RDT period, only 28.6% of the cases treated with ALu were detected later in the laboratory to be negative by microscopy compared to 78.5% of the cases treated with SP in the pre-RDT period who were later confirmed to have negative microscopy results (Table 5).

**Table 5:** Prescription of anti-malarial drugs by CORPs to febrile patients based on presence or absence of RDT results

Type of patients	Patients with BS +ve (%)	Patients treated (% )	Patients treated with RDT +ve(%)	Patients treated with RDT -ve (%)	Patients treated with BS +ve (%)	Patients treated with BS -ve (%)
**Pre-RDTs**						
Total(5576)	1130(20.5)	5478(98.6)	NA	NA	1107(20.1)‡	4319(78.5)
< 5 yrs(1065)	282(26.8)	1030(97.2)	NA	NA	260(24.8) †	750(71.6)
≥5 yrs(4511)	884(19.0)	4448(98.9)	NA	NA	838(18.8) §	3560(80.1)
**Post-RDTs**						
Total(18217)	3779(20.8)	8562(47.3)	4893(27.1)*	3629(20.1)	3383(18.7)#	5177(28.6)
< 5 yrs(4275)	850 (19.9)	4122(96.6)	951(22.4)**	3154(74.3)	826(19.4)##	3296(77.3)
≥5 yrs(13942)	2929 (21.0)	4440(32.1)	3942(28.6)***	475(3.4)	2537(18.5)###	1881(13.6)

BS = blood smear, RDT = malaria rapid diagnostic test, +ve = positive, -ve = negative, NA = not applicable

For cases with treatment records, ‡5494 in the pre-RDTs period (†1048 under-fives and §4446 aged≥5 years old) had smear results by microscopy; in the post-RDT period, *18034 cases (**4247 under-fives and ***13787 with ≥5 years old) had RDT results while #18087 (##4266 under-fives and ###13821 aged ≥5 years old) had blood smear results by microscopy; and these were used as denominators.

### Adherence to treatment guidelines by CORPs

During the pre-RDT period, 79 (1.4%) cases were not treated with anti-malarials and two had no blood smears taken while 19 (0.3%, including 11 under-fives) had malaria parasites detected later in the laboratory by microscopy. Six of the 11 under-fives were referred to the nearest dispensary for treatment while the remaining five (with geometric mean parasite density of 34,820 asexual parasites/μl) were not given a referral. Out of the remaining eight cases (≥5 years old), who were not treated by CORPs but had malaria parasites detected later by microscopy, five were referred to the nearest health facility for treatment, one had received SP treatment (in the past two weeks) and the remaining two (aged 9.3 and 36.0 years, with parasite density of 2,320 and 10,080 asexual parasites/μl, respectively) were not given a referral by CORPs. Thus, only seven cases (0.13%) went without treatment and would, therefore, remain at risk of developing severe disease in case they did not return to CORPs to seek for treatment or visit a health facility for further treatment but without a referral from CORPs.

Among children below five years of age attended during the post-RDT period, 144 (3.4%) were not treated with anti-malarials contrary to the guidelines, which required that all under-fives be treated with ALu irrespective of RDT results, giving adherence to treatment guidelines of 96.6%. For cases aged ≥5 years tested with RDTs (n = 13,787), 108(0.8%) with positive RDT results were not treated with ALu whereas 475(3.4%) with a negative RDT were treated contrary to the guidelines given to CORPs. Thus, 583(4.2%) cases were incorrectly treated by CORPs and adherence to treatment guidelines in this group was 95.8%. For those cases aged ≥5 years who were treated despite negative RDT results, only 38 (0.3%) had malaria parasites detected by microscopic examination of blood smears with geometric mean parasite density of 1,115 asexual parasites/μl (range, 40-142,000 asexual parasites/μl).

Based on blood smear results by microscopy, 249 (1.4%) cases [including eight under-fives (with geometric mean parasite density of 58,466 asexual parasites/μl; range = 40 - 394,440) and 241 cases aged ≥5 years (with geometric mean parasite density of 265; range = 40 - 160,400 asexual parasites/μl)] were neither treated with ALu nor referred to the nearest health facility by CORPs. Using a cut-off of parasite density of 5,000 asexual parasites/μl, only 36(0.2%) cases [including 5(0.1%) under-fives and 31(0.2%) aged ≥5 years] were not treated with anti-malarial drugs and thus put at risk of developing a severe disease in case they did not return to CORPs or go to find treatment elsewhere.

## Discussion

The WHO has recently recommended that treatment with anti-malarial drugs should be given to patients with malaria parasites confirmed by laboratory tests or RDTs in areas lacking the capacity for detection of malaria parasite by microscopy [9]. The Tanzanian Ministry of Health and Social Welfare, through the NMCP plans to scale-up malaria diagnosis by introducing RDTs to improve case management and reduce inappropriate dispensing of ACT, which was introduced in the country in early 2007 [14]. However, RDTs have, as also confirmed in the present study some technical and operational challenges including their accuracy and implementation. The accuracy of RDTs which is commonly measured by their sensitivity and specificity (when compared to microscopy as a gold standard) is very critical to avoid denying anti-malarial drugs to patients with malaria due to false negative results and unnecessary dispensing of drugs by treating patients with false positive RDTs. Furthermore, as recently shown [18,30,31], syndromic treatment due to lack of diagnostic facilities or non-adherence to RDT results, reduces the motivation for health-care providers to search and treat alternative causes of fever, and thus perpetuating high drug pressure which might eventually lead to parasite tolerance/resistance to ACT.

The findings of this study showed that both RDT brands (Paracheck and ParaHIT) had low sensitivity but relatively high specificity depending on fever status, parasite density, malaria parasite prevalence and transmission intensity. The overall sensitivity of RDTs was significantly higher in the longitudinal study compared to CSS and decreased with declining malaria parasite prevalence in both studies corresponding to the declining burden of malaria in the study areas as recently shown by Mmbando *et al. *[28] and Ishengoma *et al *(unpublished data). The low sensitivity observed in the CSS might mainly be due to large number of cases with low level parasitaemia, which was below the detection limits of RDTs giving rise to high rate of false negative results. The low sensitivity of RDTs observed in the current studies was similar to what has been reported in other field studies conducted in South-eastern Tanzania [21,32] and other parts with similarly low malaria endemicity [33-35]. However, it was lower than the sensitivity reported in Kilombero and north-eastern Tanzania before the recent decline in the burden of malaria [31,36] and also other parts of Africa (including East Africa, Kenya [37]and Uganda [37-39], and others [15,40,41]).

Despite the differences in parasite density in the two studies (longitudinal study and CSS) which increased with decreasing parasite prevalence, the sensitivity of RDTs was higher in febrile cases, and this was similar to what has been reported in studies conducted elsewhere in Africa [19,37,42]. Increasing parasite density with declining malaria burden observed in the study villages could be due small sample size in the recent years which led to inflated geometric mean parasite density as recently shown by other studies [28]. However, the geometric mean parasite density observed in both studies was lower than those reported in other studies using similar RDTs based on HRP-2 detection [43] and this could be the cause of relatively lower sensitivity observed in these community studies. Although the sensitivity of RDTs was higher in under-fives compared to those aged ≥5 years, the difference was not statistically significant after adjusting for other covariates. This is different from the findings reported from Kilombero, where the sensitivity of RDTs was significantly lower among older patients [36].

Despite significantly higher specificity in the CSS compared to the longitudinal study (which increased with declining parasite prevalence), the specificity observed in both studies was higher than what has been recently reported with similar RDTs (based on HRP-2) in Malawi [19]. False positive RDT results which lead to low specificity are commonly attributed to persistence of HRP-2 antigens mainly due to continued exposure to low level infections leading to sub-patent parasitaemia, gametocytaemia or delayed clearance of HRP-2 after treatment [12]. HRP-2 antigens remain in blood for over 30 days after clearance of the parasites and persistence of HRP-2 has been shown to depend mainly on parasite density at the initiation of treatment [43]. High specificity observed in the CSS indicate that most of the cases without malaria parasites residing in communities with low level of parasite prevalence will most likely be correctly diagnosed by the RDTs. However, the low specificity of RDTs among cases with fever in the longitudinal study which resulted from large number of false positive RDTs indicates that most of such cases were treated with anti-malarials despite negative results by microscopy leading to over-treatment and wastage of drugs.

Before introduction of RDTs, almost all cases attended by CORPs were treated with SP based on presenting symptoms leading to a significant level of over-treatment, since only 20.1% of all cases treated with SP during this period had malaria parasites confirmed by microscopy. However, this approach was cost-effective due to low cost of SP and was also safe since most of the febrile cases (regardless of parasite infection status) were promptly treated with anti-malarials thus reducing the risks associated with severe malaria, which often occurs when a patient is left without treatment. Despite the observed low accuracy of RDTs, which was lower than the sensitivity and specificity of ≥95% recommended by WHO [12], deployment of RDTs in the longitudinal study reduced anti-malarial dispensing by CORPs from 99% in the pre-RDT to 32% (among cases aged ≥5 years of age) in the post-RDT period. Similar studies conducted in other parts of Tanzania have recently shown that correct use of RDTs and adherence to test results by health workers reduced dispensing of anti-malarial drugs by > 60% [44]. Furthermore, majority of the cases treated with ALu during the post-RDTs period were confirmed to have malaria parasites by microscopy since only 28.6% of the cases treated had negative results by microscopy while during the pre-RDTs, 78.5% of the cases treated with SP had negative results.

Adherence to treatment guidelines by CORPs as stipulated in the SOPs was high (> 95%) in cases of all age groups. The level of adherence to treatment guidelines shown in this study was higher than what was reported by previous studies conducted in Zanzibar [45] and Burkina Faso [42,46] possibly due to the training provided to CORPs and weekly supportive supervision conducted by an experienced team. However, some cases confirmed to have malaria by microscopy (during both periods) were not treated with anti-malarials and were also not referred to the nearest health facilities for further treatment as required by the guidelines. These were relatively few (< 0.5%) and could possibly return to CORPs for treatment in case their medical conditions worsened because the services were readily available within their communities. The level and consistency of supervision has been shown to influence the proper implementation of RDTs and adherence to test results by service providers under routine health facility settings [21,32]. However, such level of supervision can hardly be provided and maintained by the health authorities when RDTs are implemented and widely used under routine clinical settings. Thus, during deployment of RDTs, health authorities will need to design and implement a sustainable scheme of supportive supervision (relevant to their local settings) to ensure the accuracy of RDTs is maintained and service providers adherence to National treatment policy and guidelines. Such strategies will also help to maintain health workers confidence on RDT results, reduce the need for syndromic treatment and target management of other causes of fevers in non-malaria cases.

The current study utilized two brands of HRP-2 based RDTs (Paracheck and ParaHIT) which showed variable results regarding their accuracy, whereby both brands had similar sensitivity in the longitudinal study while Paracheck showed higher sensitivity in the CSS. In both studies, ParaHIT had higher specificity compared to Paracheck. Although these differences could be a true reflection of their performance under field conditions in an area where malaria has remarkably declined [28], this study was not designed to address such question. The tests were used based on their availability from the NMCP and thus the study did not make any attempt to compare their performance.

Furthermore, the performance of RDTs was compared to expert microscopy whose quality cannot be attained under health facility settings as recently shown by studies conducted in Tanzania [8] and other malaria endemic areas [38]. The tests were also used for parasite detection in CSS in order to identify cases that were targeted for *in-vivo *efficacy study of anti-malarials and *in-vitro *monitoring of drug resistance (which were stopped due to lack of enough samples). Unlike in health facilities and community studies where febrile patients are commonly attended and RDTs are intended to detect patients with parasites, the tests cannot be similarly used in CSS due to low number of patients with fever, low parasite density and therefore low sensitivity as shown by the findings of the CSS. However, these findings provide important information which will potentially guide future applicability of RDTs when fully introduced in many endemic countries for malaria diagnosis in the health facilities and communities; emphasizing that the tests have more relevance for case management than for disease surveillance.

In the longitudinal study, some cases with malaria parasites (confirmed by microscopy) were neither treated with anti-malarials (SP or ALu during the pre-RDT and post-RDT periods, respectively) nor given a referral to be attended at nearby health facilities as stipulated in the guidelines given to CORPs. While it would intuitively be important to know what happened to such cases, these issues are beyond the scope of this study since only passive case detection of fever episodes was performed, and follow-up of cases after treatment or those with negative RDT results who were not given anti-malarials was not done. However, other studies conducted in northern Tanzania showed that only 1% of children with negative RDT results developed parasitaemia during the 28 days of follow-up and only 1 out of 816 children developed a severe malaria condition demanding hospitalisation (Reyburn *et al. *personal communication). Based on the setting of the longitudinal study where medical services were made available in the community through CORPs, cases not given anti-malarials had an opportunity to get treatment whenever they felt unwell, thus making this strategy safe and rational for testing the implementation of malaria case management based on RDT results.

## Conclusion

This study showed that RDTs used in the two community studies had varying accuracy (low sensitivity but relatively higher specificity) indicating that diagnosis of malaria using these HRP-2 based RDTs particularly in this era of declining burden of malaria remains a problem. Patients presenting in the health facilities from these communities for diagnosis of malaria are likely to have low parasite density which the current tests may fail to detect due to low sensitivity. High specificity of the RDTs indicates that most of the patients without malaria were correctly detected. However, introduction of RDTs in the longitudinal study reduced the number of cases without malaria who were treated with antimalarial drugs during the pre-RDTs as compared to the post-RDT period. Therefore, deployment of RDTs, coupled with supportive supervision can potentially reduce over-treatment and provide an opportunity for improved malaria diagnosis, and proper management of both malaria and non-malaria fevers. However, continued search and eventually introducing other alternative and sensitive malaria diagnostic methods should be explored.

## Competing interests

The authors declare that they have no competing interests.

## Authors' contributions

DSI, JPA, MA, TGT, IBC and MML conceived of and designed the study, DSI and PM conducted the field work and supervised the laboratory analyses. JPA and MML supervised field and laboratory work in Korogwe and Muheza districts. DSI, FF and BPM participated in data management and analysis, and DSI wrote the initial draft of the manuscript. All authors contributed during writing, read and approved the manuscript.

## References

[B1] Reyburn H New WHO guidelines for the treatment of malaria BMJ 2010 340 c2637 10.1136/bmj.c2637 20511305

[B2] O'Meara WP Mangeni JN Steketee R Greenwood B Changes in the burden of malaria in sub-Saharan Africa Lancet Infect Dis 2010 10 545 555 10.1016/S1473-3099(10)70096-7 20637696

[B3] WHO World Malaria Report 2009 2009 Geneva, Switzerland, World Health Organisation

[B4] Bates I Maitland K Are laboratory services coming of age in sub-Saharan Africa? Clin Infect Dis 2006 42 383 384 10.1086/499368 16392085

[B5] Bronzan RN McMorrow ML Kachur SP Diagnosis of malaria: challenges for clinicians in endemic and non-endemic regions Mol Diagn Ther 2008 12 299 306 18803428 10.1007/BF03256295

[B6] Petti CA Polage CR Quinn TC Ronald AR Sande MA Laboratory medicine in Africa: a barrier to effective health care Clin Infect Dis 2006 42 377 382 10.1086/499363 16392084

[B7] Ishengoma DR Rwegoshora RT Mdira KY Kamugisha ML Anga EO Bygbjerg IC Ronn AM Magesa SM Health laboratories in the Tanga region of Tanzania: the quality of diagnostic services for malaria and other communicable diseases Ann Trop Med Parasitol 2009 103 441 453 10.1179/136485909X451726 19583914

[B8] Ishengoma DR Derua YA Rwegoshora RT Tenu F Massaga JJ Mboera LE Magesa SM The performance of health laboratories and the quality of malaria diagnosis in six districts of Tanzania Ann Trop Med Parasitol 2010 104 123 135 10.1179/136485910X12607012373993 20406579

[B9] WHO Guidelines for the treatment of malaria 2010 Second Geneva, Switzerland, World Health Organization

[B10] Bell D Perkins MD Making malaria testing relevant: beyond test purchase Trans R Soc Trop Med Hyg 2008 102 1064 1066 10.1016/j.trstmh.2008.05.007 18586290

[B11] Moody A Rapid diagnostic tests for malaria parasites Clin Microbiol Rev 2002 15 66 78 10.1128/CMR.15.1.66-78.2002 11781267 PMC118060

[B12] Murray CK Gasser RA Jr Magill AJ Miller RS Update on rapid diagnostic testing for malaria Clin Microbiol Rev 2008 21 97 110 10.1128/CMR.00035-07 18202438 PMC2223842

[B13] Bell D Wongsrichanalai C Barnwell JW Ensuring quality and access for malaria diagnosis: how can it be achieved? Nat Rev Microbiol 2006 4 682 695 10.1038/nrmicro1474 16912713

[B14] Ministry of Health National Guidelines for malaria diagnosis and treatment. A.Mwita and F.Molten. -105 2006 United Republic of Tanzania, Ministry of Health and Social Welfare

[B15] Ly AB Tall A Perry R Baril L Badiane A Faye J Rogier C Toure A Sokhna C Trape JF Michel R Use of HRP-2-based rapid diagnostic test for *Plasmodium falciparum *malaria: assessing accuracy and cost-effectiveness in the villages of Dielmo and Ndiop, Senegal Malar J 2010 9 153 10.1186/1475-2875-9-153 20525322 PMC2887884

[B16] Zikusooka CM McIntyre D Barnes KI Should countries implementing an artemisinin-based combination malaria treatment policy also introduce rapid diagnostic tests? Malar J 2008 7 176 10.1186/1475-2875-7-176 18793410 PMC2556342

[B17] Bell D Malaria rapid diagnostic tests: one size may not fit all Clin Microbiol Rev 2002 15 771 772 10.1128/CMR.15.4.771-772.2002 12364379 PMC126861

[B18] Chandler CI Whitty CJ Ansah EK How can malaria rapid diagnostic tests achieve their potential? A qualitative study of a trial at health facilities in Ghana Malar J 2010 9 95 20398262 10.1186/1475-2875-9-95 PMC2859355

[B19] Chinkhumba J Skarbinski J Chilima B Campbell C Ewing V San Joaquin M Sande J Ali D Mathanga D Comparative field performance and adherence to test results of four malaria rapid diagnostic tests among febrile patients more than five years of age in Blantyre, Malawi Malar J 2010 9 209 20646312 10.1186/1475-2875-9-209 PMC2916916

[B20] Kyabayinze DJ Asiimwe C Nakanjako D Nabakooza J Counihan H Tibenderana JK Use of RDTs to improve malaria diagnosis and fever case management at primary health care facilities in Uganda Malar J 2010 9 200 10.1186/1475-2875-9-200 20624312 PMC2914063

[B21] McMorrow ML Masanja MI Abdulla SM Kahigwa E Kachur SP Challenges in routine implementation and quality control of rapid diagnostic tests for malaria--Rufiji District, Tanzania Am J Trop Med Hyg 2008 79 385 390 18784230 PMC5801444

[B22] Lusingu JP Jensen AT Vestergaard LS Minja DT Dalgaard MB Gesase S Mmbando BP Kitua AY Lemnge MM Cavanagh D Hviid L Theander TG Levels of plasma immunoglobulin G with specificity against the cysteine-rich interdomain regions of a semiconserved *Plasmodium falciparum *erythrocyte membrane protein 1, VAR4, predict protection against malarial anemia and febrile episodes Infect Immun 2006 74 2867 2875 10.1128/IAI.74.5.2867-2875.2006 16622225 PMC1459698

[B23] Mmbando BP Segeja MD Msangeni HA Sembuche SH Ishengoma DS Seth MD Francis F Rutta AS Kamugisha ML Lemnge MM Epidemiology of malaria in an area prepared for clinical trials in Korogwe, north-eastern Tanzania Malar J 2009 8 165 10.1186/1475-2875-8-165 19615093 PMC2720983

[B24] Alifrangis M Lemnge MM Ronn AM Segeja MD Magesa SM Khalil IF Bygbjerg IC Increasing prevalence of wildtypes in the dihydrofolate reductase gene of *Plasmodium falciparum *in an area with high levels of sulfadoxine/pyrimethamine resistance after introduction of treated bed nets Am J Trop Med Hyg 2003 69 238 243 14628937

[B25] Lemnge MM Msangeni HA Ronn AM Salum FM Jakobsen PH Mhina JI Akida JA Bygbjerg IC Maloprim malaria prophylaxis in children living in a holoendemic village in north-eastern Tanzania Trans R Soc Trop Med Hyg 1997 91 68 73 10.1016/S0035-9203(97)90401-6 9093633

[B26] Enevold A Alifrangis M Sanchez JJ Carneiro I Roper C Borsting C Lusingu J Vestergaard LS Lemnge MM Morling N Riley E Drakeley CJ Associations between alpha+-thalassemia and *Plasmodium falciparum *malarial infection in northeastern Tanzania J Infect Dis 2007 196 451 459 10.1086/519390 17597460

[B27] Lusingu JP Vestergaard LS Mmbando BP Drakeley CJ Jones C Akida J Savaeli ZX Kitua AY Lemnge MM Theander TG Malaria morbidity and immunity among residents of villages with different *Plasmodium falciparum *transmission intensity in North-Eastern Tanzania Malar J 2004 3 26 10.1186/1475-2875-3-26 15282030 PMC514496

[B28] Mmbando BP Vestergaard LS Kitua AY Lemnge MM Theander TG Lusingu JP A progressive declining in the burden of malaria in north-eastern Tanzania Malar J 2010 9 216 10.1186/1475-2875-9-216 20650014 PMC2920289

[B29] R Development Core Team. R A Language and Environment for Statistical Computing 2010 Vienna, Austria, R Foundation for Statistical Computing

[B30] Ansah EK Narh-Bana S Epokor M Akanpigbiam S Quartey AA Gyapong J Whitty CJ Rapid testing for malaria in settings where microscopy is available and peripheral clinics where only presumptive treatment is available: a randomised controlled trial in Ghana BMJ 2010 340 c930 10.1136/bmj.c930 20207689 PMC2833239

[B31] Reyburn H Mbakilwa H Mwangi R Mwerinde O Olomi R Drakeley C Whitty CJ Rapid diagnostic tests compared with malaria microscopy for guiding outpatient treatment of febrile illness in Tanzania: randomised trial BMJ 2007 334 403 10.1136/bmj.39073.496829.AE 17259188 PMC1804187

[B32] McMorrow ML Masanja MI Kahigwa E Abdulla SM Kachur SP Quality assurance of rapid diagnostic tests for malaria in routine patient care in rural Tanzania Am J Trop Med Hyg 2010 82 151 155 10.4269/ajtmh.2010.09-0440 20065013 PMC2803527

[B33] Bendezu J Rosas A Grande T Rodriguez H Llanos-Cuentas A Escobedo J Gamboa D Field evaluation of a rapid diagnostic test (Parascreen) for malaria diagnosis in the Peruvian Amazon Malar J 2010 9 154 10.1186/1475-2875-9-154 20529273 PMC2898785

[B34] Mens P Spieker N Omar S Heijnen M Schallig H Kager PA Is molecular biology the best alternative for diagnosis of malaria to microscopy? A comparison between microscopy, antigen detection and molecular tests in rural Kenya and urban Tanzania Trop Med Int Health 2007 12 238 244 17300631 10.1111/j.1365-3156.2006.01779.x

[B35] Sayang C Soula G Tahar R Basco LK Gazin P Moyou-Somo R Delmont J Use of a histidine-rich protein 2-based rapid diagnostic test for malaria by health personnel during routine consultation of febrile outpatients in a peripheral health facility in Yaounde, Cameroon Am J Trop Med Hyg 2009 81 343 347 19635896

[B36] Laurent A Schellenberg J Shirima K Ketende SC Alonso PL Mshinda H Tanner M Schellenberg D Performance of HRP-2 based rapid diagnostic test for malaria and its variation with age in an area of intense malaria transmission in southern tanzania Malar J 2010 9 294 10.1186/1475-2875-9-294 20974009 PMC2974751

[B37] Abeku TA Kristan M Jones C Beard J Mueller DH Okia M Rapuoda B Greenwood B Cox J Determinants of the accuracy of rapid diagnostic tests in malaria case management: evidence from low and moderate transmission settings in the East African highlands Malar J 2008 7 202 10.1186/1475-2875-7-202 18834523 PMC2571107

[B38] Batwala V Magnussen P Nuwaha F Are rapid diagnostic tests more accurate in diagnosis of *Plasmodium falciparum *malaria compared to microscopy at rural health centres? Malar J 2010 9 349 10.1186/1475-2875-9-349 21126328 PMC3002380

[B39] Hopkins H Bebell L Kambale W Dokomajilar C Rosenthal PJ Dorsey G Rapid diagnostic tests for malaria at sites of varying transmission intensity in Uganda J Infect Dis 2008 197 510 518 10.1086/526502 18240951

[B40] Gerstl S Dunkley S Mukhtar A De Smet M Baker S Maikere J Assessment of two malaria rapid diagnostic tests in children under five years of age, with follow-up of false-positive pLDH test results, in a hyperendemic falciparum malaria area, Sierra Leone Malar J 2010 9 28 10.1186/1475-2875-9-28 20092620 PMC2835716

[B41] Singh N Shukla MM Shukla MK Mehra RK Sharma S Bharti PK Singh MP Singh A Gunasekar A Field and laboratory comparative evaluation of rapid malaria diagnostic tests versus traditional and molecular techniques in India Malar J 2010 9 191 10.1186/1475-2875-9-191 20602766 PMC2905433

[B42] Bisoffi Z Sirima SB Menten J Pattaro C Angheben A Gobbi F Tinto H Lodesani C Neya B Gobbo M Van den EJ Accuracy of a rapid diagnostic test on the diagnosis of malaria infection and of malaria-attributable fever during low and high transmission season in Burkina Faso Malar J 2010 9 192 10.1186/1475-2875-9-192 20609211 PMC2914059

[B43] Kyabayinze DJ Tibenderana JK Odong GW Rwakimari JB Counihan H Operational accuracy and comparative persistent antigenicity of HRP2 rapid diagnostic tests for Plasmodium falciparum malaria in a hyperendemic region of Uganda Malar J 2008 7 221 10.1186/1475-2875-7-221 18959777 PMC2584069

[B44] D'Acremont V Kahama-Maro J Swai N Mtasiwa D Genton B Lengeler C Reduction of anti-malarial consumption after rapid diagnostic tests implementation in Dar es Salaam: a before-after and cluster randomized controlled study Malar J 2011 10 107 10.1186/1475-2875-10-107 21529365 PMC3108934

[B45] Msellem MI Martensson A Rotllant G Bhattarai A Stromberg J Kahigwa E Garcia M Petzold M Olumese P Ali A Bjorkman A Influence of rapid malaria diagnostic tests on treatment and health outcome in fever patients, Zanzibar: a crossover validation study PLoS Med 2009 6 e1000070 10.1371/journal.pmed.1000070 19399156 PMC2667629

[B46] Bisoffi Z Gobbi F Angheben A Van den EJ The role of rapid diagnostic tests in managing malaria PLoS Med 2009 6 e1000063 10.1371/journal.pmed.1000063 19399160 PMC2667642

